# Evaluation of Crystal Zenith Microtiter Plates for High-Throughput Formulation Screening

**DOI:** 10.1016/j.xphs.2019.10.027

**Published:** 2020-01

**Authors:** J. Alaina Floyd, Jeremy M. Shaver, Alison J. Gillespie, Unjy Park, Richard S. Rogers, Nancy S. Nightlinger, Yuko Ogata, Jeffrey J. James, Bruce A. Kerwin

**Affiliations:** Just Biotherapeutics, Inc., Seattle, Washington 98109

**Keywords:** antibody(s), automation, developability screening, formulation, high throughput technology(s), protein(s), stability

## Abstract

Formulation screening for biotherapeutics can cover a vast array of excipients and stress conditions. These studies consume quantities of limited material and, with higher concentrated therapeutics, more material is needed. Here, we evaluate the use of crystal zenith (CZ) microtiter plates in conjunction with FluoroTec-coated butyl rubber mats as a small-volume, high-throughput system for formulation stability studies. The system was studied for evaporation, edge effects, and stability with comparisons to type 1 glass and CZ vials for multiple antibodies and formulations. Evaporation was minimal at 4°C and could be reduced at elevated temperatures using sealed, mylar bags. Edge effects were not observed until 12 weeks at 40°C. The overall stability ranking as measured by the rate of change in high molecular weight and percent main peak species was comparable across both vials and plates at 4°C and 40°C out to 12 weeks. Product quality attributes as measured by the multi-attribute method were also comparable across all containers for each molecule formulation. A potential difference was measured for subvisible particle analysis, with the plates measuring lower particle counts than the vials. Overall, CZ plates are a viable alternative to traditional vials for small-volume, high-throughput formulation stability screening studies.

## Introduction

During the development of biotherapeutics such as monoclonal antibodies (mAbs) or Fc-fusion proteins, the establishment of a stable formulation for the drug product can be both time- and material-consuming.^1^^,^^2^ Formulation stability screening can cover a vast array of pH, buffer, excipient, and surfactant conditions in addition to a variety of stress conditions such as elevated temperature, mechanical agitation, and light exposure to determine the chemical, physical, and colloidal stability of the molecule.^1^^,^^3^ Each of these studies consumes formulated material and with the increasing trend of using higher concentration therapeutics to minimize injection volume,^4^ more material is necessary for such studies.

While final stability studies are conducted in a drug product configuration such as vials or pre-filled syringes, the preliminary formulation screening studies can be conducted on a smaller scale using microtiter plates. This type of format is advantageous because of its small footprint (a 96-well plate is about 128 mm long by 85 mm wide^5^) and the use of small volumes in the wells. Already, microtiter plates are used in high-throughput biophysical stability screening such as differential scanning fluorimetry, spectroscopy including UV-Vis absorbance, luminescence, and fluorescence, size-exclusion chromatography (SEC), and chemical unfolding.^1^^,^^3^^,^^6^, ^7^, ^8^, ^9^ However, there are limited examples in the literature concerning long-term stability studies in microtiter plates.^1^^,^^7^^,^^10^

One such example is a study by Alekseychyk et al.^1^ that used SEC to monitor the stability of mAbs in hard-shell, full-skirted PCR plates out to 2 weeks at 4°C, 25°C, and 40°C. While differentiation between formulations was achieved, there was no direct comparison to vials to indicate whether these data were representative. Casaz et al.^10^ also used SEC to study antibodies in polystyrene microtiter plates at 4°C and 37°C out to 4 weeks. When the long-term, 6-month, 37°C percent dimer data in glass vials were compared to the short-term plate data across 3 formulations, there was good agreement on 2 of the formulation rankings, with the phosphate-based formulation being the least stable and the acetate formulation being most stable. However, the plates predicted that the citrate formulation would behave more similarly to the phosphate formulation whereas the long-term stability in vials showed the citrate formulation to be more similar to the acetate formulation. One interpretation is that this discrepancy could arise from the reported evaporation loss, protein interaction with the polystyrene plates, or the different buffer exchange methods and final protein concentration that was used between the plate and vials.

While the use of commercially available microtiter plates composed of polystyrene, polypropylene, and other plastics is attractive for cost and availability reasons, they have the drawbacks of potential material loss due to the protein nonspecifically adsorbing to the surface, the risk of leachables and extractables from the plastic, and potential for aggregation from surface adsorption-desorption.^11^, ^12^, ^13^, ^14^, ^15^, ^16^ Glass inserts that are in a 96-well plate format can be a way around plastic-protein interaction and have been previously studied but have the drawback of high cost.^7^

**Figure 1:**
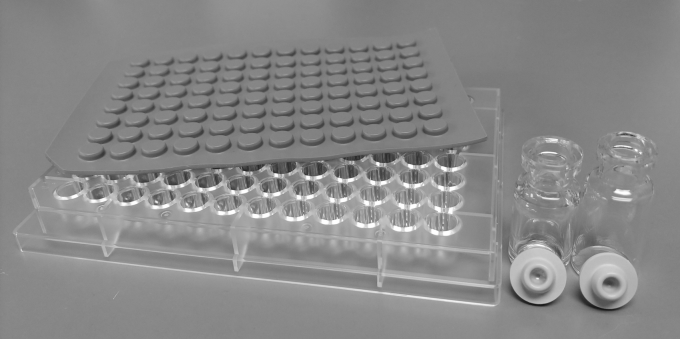
From left to right, an example of a CZ plate, a CZ vial, and a glass vial.

A new type of 96-well, microtiter plates that has not been previously studied for biotherapeutic formulation stability studies are plates composed of Crystal Zenith® (CZ). CZ is a cyclic olefin polymer that is commercially available in a vial format as an alternative to traditional glass vials. CZ vials have been shown to perform well at extreme cold conditions (−80°C and −196°C), are more resistant to breakage than glass, and are not prone to delamination as are glass vials.^17^ In some cases, CZ vials have been shown to have lower protein binding than traditional glass vials.^18^^,^^19^ CZ vials also have a well-studied leachable and extractable profile and have been approved by several global regulatory agencies as packaging for specific therapeutics.^16^^,^^18^^,^^20^^,^^21^ CZ plates follow ANSI dimension standards and are provided with a plug-mat that is composed of butyl rubber with a FluoroTec® coating (Fig. 1). This type of coating is preferred to plain, butyl rubber stoppers because the FluoroTec coating has been postulated to prevent leachates from rubber stoppers that have been previously linked to immunogenic effects.^22^ This coating also matches coatings that are commonly in use for stoppers for glass vials.

A method to monitor whether proteins are affected by using a CZ microtiter plate instead of a more traditional glass or CZ vial is to use the multi-attribute method (MAM). MAM is a mass spectrometry–based method that can monitor known product quality attributes and detect potential impurities.^23^^,^^24^ MAM consists of 2 components: attribute analytics and new peak detection. Attribute analytics is the relative quantification of a known product quality attribute (PQA). New peak detection (NPD) is a comparison of a test sample to a reference standard. NPD is accomplished by leveraging software that compares every peak in the test sample to the reference standard. Using these 2 components, microtiter plates can be tested against the standard glass vials to determine whether new peaks are detected and whether certain product quality attributes are changing based on the container or are uniform across containers.

Evaporation can also be a problem for microtiter plates and the type of seal/mat must be carefully chosen. In a study by Casaz et al.,^10^ the volume in the inner 60 wells of a 96-well, polystyrene plate decreased by 6%-10% after 30 days at 37°C. Evaporative losses were greater along the plate edges, 10%-15%, and up to 20% in the plate corners. These types of losses correspond to an increasing concentration of protein and excipients, all of which can impact stability and do not model the conditions of the drug in either a vial or syringe. Vials are expected to have no evaporative loss and are tested for container closure integrity. The well-fitting rubber stopper and crimp seal both contribute to the integrity of the closure. This crimp seal is not available for microtiter plates, so it is important to test for evaporative loss and find conditions that minimize the problem.

In this study, we examine the evaporative loss from 96-well plates composed of CZ with the FluoroTec-coated, butyl rubber mat at low and elevated storage temperatures. SEC and reduced capillary electrophoresis-sodium dodecyl sulfate (rCE-SDS) are used to test for any edge or position effects across the entirety of a plate at 40°C over 12 weeks for 2 different formulations. Furthermore, multiple molecules (3 mAbs and 1 Fc-fusion protein) and multiple formulations are compared using SEC and rCE-SDS from material stored in a traditional glass vial, a CZ vial, and a CZ plate. MAM analysis is employed to determine the presence of new peaks and similarity in degradation trends between the 3 configurations for select molecules and formulations. Finally, subvisible particle formation is studied for 2 formulations across the 3 container types.

## Materials and Methods

### Materials

Water (MilliQ, 18.2 μΩ), Roche LightCycler 480 sealing foil (PN 04729757001), mylar bags (6 × 8″ 2.5 MIL, U-Line), heat sealer (Poly Bag Sealer, 8” Impulse Sealer, U-Line), crimp caps (West, PN 54130341), glass vials (Schott, PN 1542306, sterilized by ethylene oxide), stoppers (Daikyo, PN S2-F451 RUV), CZ vials (West, PN 26017611, e-beam sterilized), the Protein Express Assay Reagent Kit (Perkin Elmer), and β-mercaptoethanol (Sigma, ≥99%) were used as received. CZ 96-well plates (Daikyo) and mats (Daikyo, FluoroTec coated) were autoclaved before use. Reagents used in MAM are as described by Rogers et al.^23^

The Fc-fusion protein and mAbs 1, 2, and 3 were produced using stable CHO-K1 cell lines and purified using standard protein A and polishing chromatography. Material was formulated into the conditions listed in Table 1.

**Table 1:** List of Protein Samples and Formulation Conditions, ^a^Analyzed by MAM

Protein Type	Formulation	Formulation Acronym
mAb 1	20 mM Acetate, 9% sucrose wt/v, polysorbate 80 0.01% wt/v, pH 5.2, 100 mg/mL	A52SuT
	20 mM Acetate, 3% proline wt/v, polysorbate 80 0.01% wt/v, pH 5.2, 150 mg/mL	A52ProT^a^
	20 mM Acetate, 3% proline wt/v, pH 5.2, 150 mg/mL	A52Pro
mAb 2	20 mM Acetate, 9% sucrose, wt/v, polysorbate 80 0.01% wt/v, pH 5.2, 33 mg/mL	A52SuT^a^
mAb 3	20 mM Acetate, 9% sucrose wt/v, polysorbate 80 0.01% wt/v, pH 5.2, 90 mg/mL	A52SuT^a^
Fc-fusion protein	10 mM Phosphate, 40 mM NaCl, 5% sucrose wt/v, 0.03% polysorbate 20 wt/v, pH 6.2, 40 mg/mL	P62NaSuT^a^
	10 mM Acetate, 3% proline wt/v, 0.1% Pluronic F68 wt/v, pH 5.2, 40 mg/mL	A52ProPl
	10 mM Acetate, 3% proline wt/v, 0.03% polysorbate 80 wt/v, pH 5.2, 40 mg/mL	A52ProT
	10 mM Phosphate, 2.75% lysine wt/v, 0.1% Pluronic F68 wt/v, pH 6.2, 40 mg/mL	P62LysPl^a^
	10 mM Phosphate, 2.75% lysine wt/v, 0.03% polysorbate 80 wt/v, pH 6.2, 40 mg/mL	P62LysT
	10 mM phosphate, 3% proline wt/v, 0.1% Pluronic F68 wt/v, pH 6.2, 40 mg/mL	P62ProPl
	10 mM Phosphate, 3% proline wt/v, 0.03% polysorbate 80 wt/v, pH 6.2, 40 mg/mL	P62ProT

### Microtiter Plate Volume Measurements

Microtiter plate volume measurements were completed using a BioMicroLab VolumeCheck 50. An initial calibration table was created using a CZ plate and known volumes of water ranging from 0 to 0.2 mL. Before measurement, each plate was spun down at 3500 rpm for 4 min. The plates were then briefly tapped on a flat surface to ensure that the fluid was centered at the bottom of the well. Each well of the plate was scanned 3 times and the average volume and standard deviation reported.

### Water Evaporation From the CZ Plate

To each well of the CZ plate, 0.1 mL of water was added. After centrifugation, each plate was measured on the VolumeCheck 50 as a time zero reference. Each plate was then sealed using the FluoroTec-coated rubber mat and placed at −70°C, −30°C, 4°C, 30°C, or 40°C. Select plates were placed in a mylar bag containing 1 mL of water in the bottom and then sealed using 2, parallel heat seals before being placed at their respective temperatures. After incubation, plates were removed, centrifuged, and tapped down before being measured. Results are reported as the total percent volume remaining after incubation.

### Size-exclusion High Performance Liquid Chromatography

SEC was performed on a Dionex UltiMate 3000 HPLC System using a Waters XBridge Protein BEH SEC 200Å, 3.5-μm column and a diode array detector collecting at 280 nm. Separation for mAb 1 and 3 and the Fc-fusion protein was achieved under native conditions with a 100 mM sodium phosphate, 250 mM sodium chloride mobile phase buffer at pH 6.8. MAb 2 was analyzed with a 10% acetonitrile v/v, 100 mM sodium phosphate, and 250 mM sodium chloride mobile phase buffer at pH 6.8. Results were analyzed using an in-house tool that automatically aligns and chooses peak regions to integrate. The alignment removes time-axis variations using a constrained version of correlation optimized warping,^25^ in which the detected shifts in each window are robustly fit to a linear function. Integration regions are then selected by automatically identifying the discrete valley minimum between each peak response where a peak-to-valley height was larger than a noise threshold. For SEC, the raw response versus time was used to determine the peak and valley locations.

### Reduced Capillary Electrophoresis-Sodium Dodecyl Sulfate

rCE-SDS was performed on a PerkinElmer LabChip® GX II Touch HT using fluorescence detection. The LabChip was prepared as recommended by PerkinElmer’s Protein Express Assay Quick Guide using the Protein Express Assay Reagent Kit.^26^ Samples were diluted to 1 mg/mL in water either by hand or using a liquid handling robot (Tecan Freedom Evo). Now 14 μL of a 3.5% 2-mercaptoethanol solution in water (v/v) was added to 4 μL of the diluted samples. Samples were denatured at 70°C for 10 min, then diluted with 70 μL of water before being analyzed by the LabChip. Results were analyzed using the same in-house tool as SEC except a sharpened version of each trace was created and used to locate peak and valley locations. The sharpened trace was created by adding the raw trace to a scaled copy of the second derivative of the given trace. This emphasizes inflection points and helps detect peak shoulders. The mid-molecular weight (MMW) is the percent area of the peaks measured between the light and heavy chain (HC) for a mAb and between the nonglycosylated HC and main for the Fc-fusion. The low molecular weight (LMW) is the percent area of the peaks measured before a light chain for a mAb and before the nonglycosylated HC for the Fc-fusion.

### Subvisible Particle Analysis

Subvisible particle analysis was performed on a Fluid Imaging FlowCam and ALH system. Samples were mixed, aliquoted in a 96-well plate (Cellstar, PN 650970), and measured using the ALH system. An amount of 50 μL was measured for each sample replicate. For the 12-week stability study, 3 replicates were analyzed for the glass and CZ vials. Only 2 replicates were analyzed for the plate during the stability study due to sample volume limitation. Tergazyme and Milli-Q water were used to clean the flow cell in between each sample. A 7-day study at room temperature was conducted with 2 mL of mAb 3 in a CZ and glass vial and 63 μL in each well of the CZ plate. Samples were measured in triplicate out of the vials and CZ plate immediately after pipetting mAb 3 into the container, 24 h later, and 7 days later.

### Multi-attribute Method

Samples were prepared as described by Rogers et al.^23^

### Reversed-phase Liquid Chromatography for MAM

A dual column Vanquish Flex Binary UPLC was used (Thermo). Mobile phase A contained 0.1% formic acid in water and mobile phase B contained 0.1% formic acid in 100% acetonitrile. The following liquid chromatography conditions were used for separating the peptides: flow rate 0.3 mL/min, column temperature 50°C during the separation, and the auto-sampler was kept at 4°C. For each analysis, a nominal load of 2 μg of the digest, based on final sample concentration, was injected onto a Zorbax C18 300-SB, 300 Å pore size, 1.8-μm particle, 2.1 mm × 150 mm column (Agilent). The gradient started at 2% B until 5 min, then increased gradually to 10% in 1 min. Next, the gradient was ramped up from 10% B to 35% B in 44 min. Next, the %B is increased to 60% in 5 min. At 55 min, the %B is dropped to 2% for 5 min. The total method time is 60 min. As the next sample is injected, the valve on the column heater switches to use the second column. The first column is washed with saw tooth gradients (2% B to 90% B and back down) until minute 45. From minute 45 to 60, 2% B is used.

### Mass Spectrometry Conditions for MAM

The tryptic peptides were separated and monitored by RP-HPLC coupled to MS (Thermo Q Exactive HF). The MS capillary temperature was maintained at 250°C with an S-lens RF level at 50. The MS spectra collection was performed at 120,000 resolution in positive polarity mode with an AGC target of 3E6, maximum ion time of 200 ms, and a scan range of 300 to 1800 m/z between 5 and 55 min run time.

### Search Parameters for MAM

Biopharma Finder 3.1 (Thermo) was used to identify the peptides using the default variable modifications and the CHO glycan library. A static modification for all cysteines was used (+58.005, carboxymethylation) and unspecified modifications were allowed between −58 and 162. New peak detection was performed using Biopharma Finder 3.1 (Thermo). A reference sample was set in the method and all other files were compared to that reference. An intensity threshold of 5e5 and a fold change of 10 was used to filter the data for NPD.

### Plate Positional Effects at 40°C

Two formulations of the Fc-fusion protein, P62NaSuT and A52ProPl, were used to determine the presence of positional or edge effects across the CZ plate at an elevated temperature. A 0.1 mL of the Fc-fusion protein was added to the inner 60 wells of 3 CZ plates per formulation. And 0.1 mL of water was added to the remaining 36 outer-border wells. The plates were sealed with the mat and the mylar bag method before being placed at 40°C for 4, 8, or 12 weeks. For comparison, an additional 0.3 mL was placed in 3 glass vials per formulation, stoppered, crimped sealed, and placed at 40°C for 4, 8, or 12 weeks. At each time point, 1 plate and 1 vial from each formulation was removed and analyzed by SEC and rCE-SDS.

### Comparison of CZ Plate, CZ Vial, and Glass Vial at 4°C and 40°C Over Time

Three mAbs and 1 Fc-fusion protein were formulated as described in Table 1. For each formulation, 0.5 mL was filled into 6 CZ vials, 6 glass vials, and 0.1 mL was added to 5 wells of the inner 60 wells of 6 CZ plates. Then, 0.1 mL of water was added to the 36 outer-border wells. All vials were stoppered and crimp sealed. Three of the CZ plates were sealed with just the mat whereas 3 of the plates were sealed with the mat and the heat-sealed mylar bag method. One CZ and glass vial and 1 CZ plate were placed at 4°C and 40°C for 4, 8, or 12 weeks. The mylar-sealed plates were placed at 40°C whereas the mat-sealed plates were placed at 4°C. At each time point, 1 plate and 1 of each vial type were removed and analyzed by SEC, rCE-SDS, and for select formulations, MAM and subvisible particle analysis.

## Results and Discussion

A major drawback when using a microtiter plate at elevated temperatures, as is the case for accelerated stability studies, is the loss of material volume from the plate due to evaporation. As the solution evaporates, the protein becomes more concentrated which can impact the stability profile of the molecule. This becomes more important at higher stability temperatures such as 40°C. In a worst-case scenario, a poor seal can allow for the complete evaporation of the sample, thereby negating the study.

**Figure 2:**
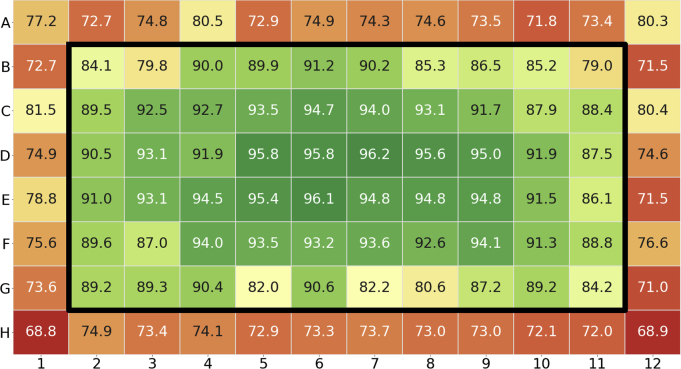
The percent volume remaining in a CZ plate after storage at 30°C for 6 weeks. The heavy black line surrounds the inner 60 wells.

The CZ plates were initially studied to determine the evaporative losses of water at 30°C and 40°C, common temperatures for accelerated stability studies. However, when the coated mat and plate were examined at elevated temperatures, clear evaporative edge effects were measured. For example, Figure 2 shows the percent volume remaining across the entire plate after 6 weeks at 30°C. Most of the loss occurred along the outer edge of the plate. When the outer 36 wells were removed and treated as a sacrificial water barrier, the percent volume remaining increased from 84.3% ± 8.8% to 90.5% ± 4.3%. Casaz et al.^10^ also reported similar effects in a multi-well, polystyrene plate and used the edge wells as a water barrier to slow evaporation. Notably, their reported evaporative loss after 30 days at 37°C was 6%-10% from the plate which is comparable to the CZ plate performance of ~10% volume loss after 4 weeks (28 days) at 40°C. Because this edge effect was noticed in all plates at elevated temperatures, the outer wells were treated as a buffer and only the inner 60 wells were used at elevated temperatures.

**Figure 3:**
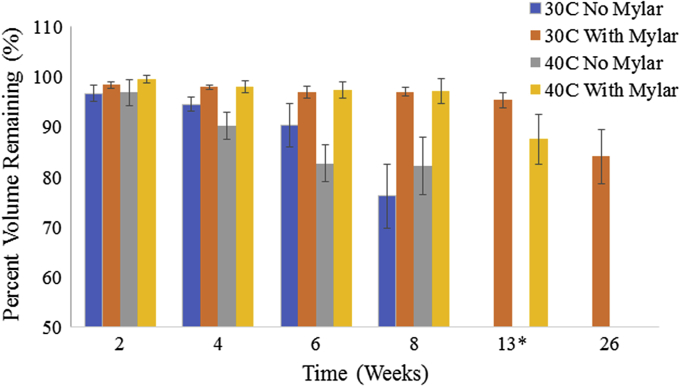
Evaporation from CZ plates with and without a mylar bag seal at 30°C and 40°C. Volumes are based on the inner 60 wells. Due to high evaporative losses, plates without mylar bags were not measured at the 13-week time point and only the 30°C condition with the mylar bag was measured at 26 weeks *The 40°C condition was measured at 12 weeks, 30°C at 13 weeks.

To minimize the evaporation from the CZ plates at elevated temperatures, the use of a mylar bag to seal the plate was introduced. Mylar bags are commonly used for long-term food storage in conjunction with food grade containers. The mylar layer helps prevent passage of nitrogen, oxygen, carbon dioxide and other gasses, water vapor, and light.^27^^,^^28^ Additionally, mylar bags that are Food and Drug Administration and the United States Department of Agriculture compliant are commercially available. Figure 3 shows the water evaporation results at 30°C and 40°C with and without the mylar bag. The use of the mylar bag significantly reduces the rate of evaporation, enabling the 30°C and 40°C to last long term with minimal evaporative effects, approximately 5% and 16% volume loss (averaged across the inner 60 wells) after 13 and 26 weeks at 30°C, respectively, and approximately 12% loss after 12 weeks at 40°C. Without the mylar bag, the plate has greater than 10% loss at 8 and 6 weeks for 30°C and 40°C, respectively. An additional plate was measured at 40°C after 12 weeks with the mylar bag to confirm consistency in the evaporation loss. The 2 plates were statistically the same, 87.6% ± 5.0% and 84.4% ± 4.4% volume remaining.

**Figure 4:**
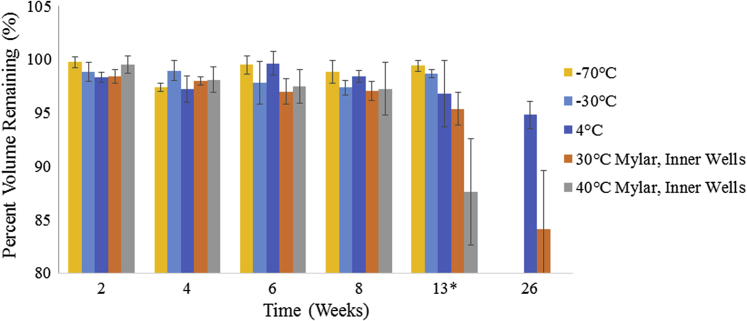
Evaporation from CZ plates across multiple temperatures and storage lengths. The 30°C and 40°C plates used the mylar bag seal method and report only the inner 60 wells whereas the −70°C, −30°C, and 4°C temperatures do not use the mylar bag and report the percent volume remaining across all wells. *−70°C, 4°C, and 30°C were measured at 13 weeks, −30°C at 14 weeks, and 40°C at 12 weeks.

Other common stability storage temperatures were studied without the use of a mylar bag and are summarized in Figure 4. At −70°C and −30°C, a negligible evaporative loss is detected, less than 2% across all 96 wells of the plate out to 13 and 14 weeks, respectively. It is hypothesized that this trend would continue at these freezing temperatures for longer durations, potentially up to a year or 2 years based on the negligibly changing volumes out to 3 months. At 4°C, there is a slight increase in evaporative loss; however, only 5.2% of the volume was lost across the entire plate after 26 weeks of storage. Due to the low volume loss at these temperatures, the entirety of the plate is used without a water buffer zone that is necessary at 30°C and 40°C.

**Figure 5:**
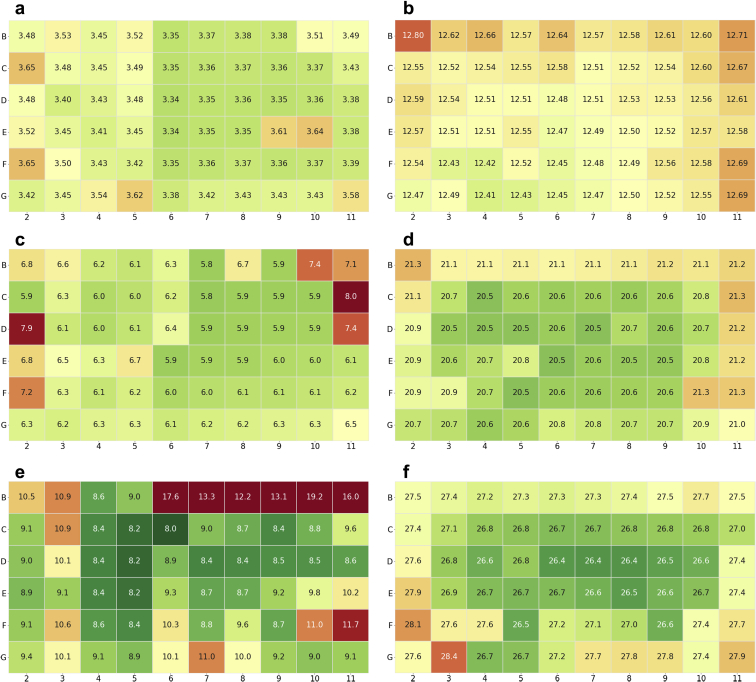
SEC results for the Fc-fusion protein across the inner 60 wells of a CZ plate in A52ProPl after (a) 4, (c) 8, and (e) 12 weeks and P62NaSuT after (b) 4, (d) 8, and (f) 12 weeks at 40°C. The color scale for (e) has been limited to 8%-12% to better show gradations.

While the evaporation from the plate at 40°C with the mylar bag was minimal, there was concern that evaporative effects could still influence stability measurements made by SEC and rCE-SDS. To assess this, 2 control samples were created in which a single molecule was plated across the entire inner 60 wells of a plate and in a glass vial and stored at 40°C for 4, 8, and 12 weeks. This was done with both a stabilizing and nonstabilizing formulation, as assessed by protein high-molecular-weight (HMW) levels. Table 2 lists the averaged HMW results for both formulations and configurations, and Figure 5 shows the individual HMW results for both formulations across the plate at each time point. Similar results were obtained for the glass vial and plate at the 4- and 8-week time point. A greater difference was measured at the 12-week time point. While at 4 and 8 weeks, the greater HMW values are occurring along the border edges without a set pattern, the range of values measured are very narrow as reflected by the low standard deviation (Figs. 5a-5d). However, at 12 weeks at 40°C, there is a noticeable edge effect in the HMW data. For P62NaSuT, the greater HMW values are consistently along the edge whereas for A52ProPl, there are 6 wells along the edge with a significantly greater HMW, perhaps from a poor seal (Figs. 5e and 5f). The increased HMW along the edges correlates with the water evaporation data, where there is approximately a 12% decrease in volume at 40°C, with the greatest loss occurring along the edges of the plate. The increased evaporation causes the protein in the well to become more concentrated. As the concentration increases so does the frequency of protein-protein interaction, leading to more chances for stable aggregates to form, potentially explaining why there is more HMW along the edges of the plate at 40°C after 12 weeks.^29^, ^30^, ^31^ To confirm evaporation of the formulation buffer, the protein concentration was calculated using the raw (vs. relative) area under the curve and compared to the calculated concentration in the glass vial at the corresponding condition. The evaporation from the protein-formulation buffer followed the same trend as the water evaporation, approximately 95% and 94% volume remaining after 4 weeks at 40°C and approximately 86% and 84% volume remaining after 12 weeks at 40°C for A52ProPl and P62NaSuT, respectively.

**Table 2:** Averaged %HMW From SEC Across the Inner 60 Wells of a CZ Plate Compared to a Glass Vial at 40°C Over Time

Formulation	Configuration	4 wk	8 wk	12 wk
A52ProPl	CZ plate	3.44% ± 0.08%	6.29% ± 0.48%	9.83% ± 2.16%
	Glass vial	3.55%	6.47%	8.47%
P62NaSuT	CZ plate	12.55% ± 0.07%	20.80% ± 0.26%	27.11% ± 0.49%
	Glass vial	12.19%	20.54%	27.76%

The percent main, MMW, and LMW as measured by rCE-SDS were also measured at these time points across the entirety of the plate. Table 3 lists the averaged values across the plate and the value measured from the glass vial control. When the average plate values are compared to the glass vials, the values are comparable at 4 weeks. At 8 weeks, there is a slight difference between the plate and vial, the percent main is about 2% lower in the vial than the plates. This corresponds to a higher value for the percent MMW and LMW in the glass vial compared to the plate. For A52ProPl at 12 weeks, this trend persists but the difference is reduced to about 1%. For P62NaSuT at 12 weeks, there is a larger difference in the plate and vial, with the vial being about 5% greater in percent main than the plate, with corresponding lower values for percent MMW and LMW in the vial.

**Table 3:** rCE-SDS Results for A52ProPl and P62NaSuT Across the Inner Wells of the CZ Plate Compared to the Glass Vial Control After 4, 8, and 12 Weeks at 40°C

Formulation	Configuration	rCE Results	4 wk	8 wk	12 wk
A52ProPl	CZ plate	%Main	84.7 ± 0.1	82.36 ± 0.3	74.1 ± 0.3
		%MMW	4.3 ± 0.1	3.30 ± 0.1	7.2 ± 0.1
		%LMW	2.7 ± 0.1	3.41 ± 0.1	6.2 ± 0.0
	Glass vial	%Main	84.7	80.3	73.3
		%MMW	4.4	3.9	8.4
		%LMW	2.7	4.3	6.4
P62NaSuT	CZ plate	%Main	86.4 ± 0.4	84.2 ± 0.1	75.4 ± 1.1
		%MMW	3.6 ± 0.1	2.69 ± 0.1	3.94 ± 0.1
		%LMW	2.3 ± 0.1	2.76 ± 0.1	6.71 ± 0.9
	Glass vial	%Main	86.6	82.4	80.3
		%MMW	3.6	3.4	3.2
		%LMW	2.3	3.3	4.4

**Figure 6:**
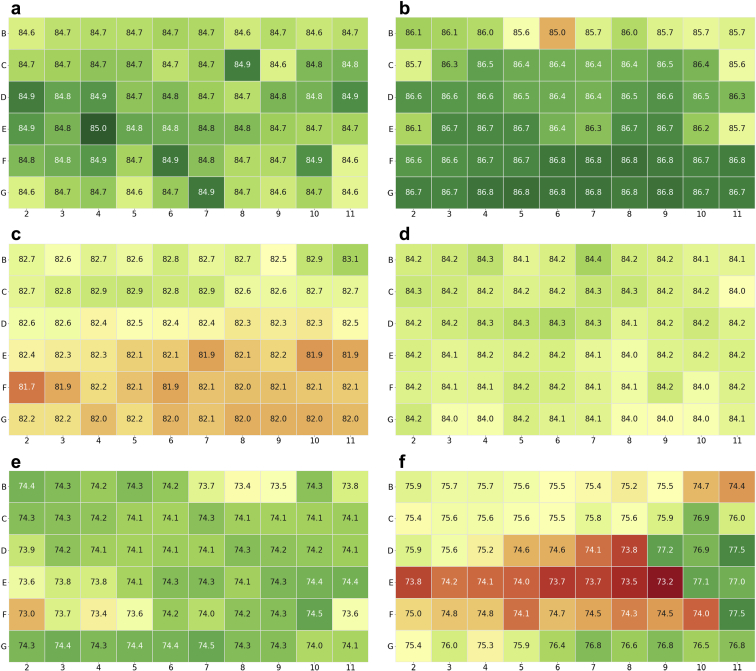
The individual well results for percent main from rCE-SDS: (a), (c), and (e) are A52ProPl at 4, 8, and 12 weeks, respectively, whereas (b), (d), and (f) are P62NaSuT at 4, 8, and 12 weeks, respectively.

Figure 6 shows the individual well results for percent main for both formulations at each time point. At 4 weeks, there is a noticeable difference between the A52ProPl and P62NaSuT pattern of results across the plate. The A52ProPl results appear random across the plate whereas the P62NaSuT results appear to follow a row format. At this time point, the samples were diluted by hand using a multi-channel pipette, pipetting by row. It is hypothesized that this dilution caused the row trend in the results. To avoid the influence of manual bias, all dilutions were made by a liquid handling robot at the 8- and 12-week time points and the row effect is no longer observed. At the 8-week time point, there does not appear to be edge effects or any continuation of a pattern.

The strong edge effects that were measured by SEC at 12 weeks are not apparent in the rCE-SDS results at 12 weeks. Instead, there are sections of the P62NaSuT and A52ProPl formulations that are varying. This variation was stronger in the P62NaSuT plate, causing a wider standard deviation than previous time points. It is hypothesized that this variation in the rCE-SDS results may have arisen from poor mixing in the well rather than from evaporative effects. There was not a strong mixing protocol in effect for sampling from the plate for rCE-SDS measurements. As such, settlement of protein may have occurred, and the resultant nonhomogeneous mixture could have produced the variation measured at 12 weeks. To confirm that this variation was not within the error of the assay, a sample was measured repeatedly (30 times) on rCE-SDS. The variation, as measured by the standard deviation for the different components from the repeat measurements, was less than 0.1%. This indicates that the differences measured for the main peaks are indicative of sample differences and not instrument variability.

Taking both the rCE-SDS and SEC measurements into account, all 60 wells of the CZ plates are following the same trend as the glass vials with some slight variation after 4 and 8 weeks at 40°C. As such, it would be strongly recommended that multiple samples be used in a plate to deal with any random variation. However, after 12 weeks at 40°C, there is a larger deviation measured across the wells and between the plates and glass vial. Approximately 14%-16% of the volume in the wells have evaporated at this time point, with more evaporative loss occurring along the edge. This edge effect is the likely cause for the larger standard deviations and differences between the plate and glass vial. As such, the CZ plates should be used cautiously for any studies going out to or past the 12-week time point at 40°C. Options to overcome this limitation are to use only the inner 32 wells to avoid edge effects, having a large sample size per condition, or creating a CZ plate that has water channels built into the walls and between the wells of the plate to minimize evaporation.

With the CZ plates demonstrating minimal plate positional effects at the 4- and 8-week time points at 40°C, a larger, multi-molecule and multi-formulation stability study was designed to further test the robustness of the plates. In this study, the CZ plates are compared to traditional, commercially available glass and CZ vials over 4, 8, and 12 weeks at 4°C and 40°C. Five replicates were used per sample to account for variation due to evaporation at the 12-week time point. Additionally, the rate of change was calculated and used for evaluation criteria as it reduces the impact of the potential error at the 12-week time point by using all 4 time points (T0-12 weeks). Three different mAbs and an Fc-fusion protein were studied with a range of pH and surfactant compositions to ensure the effects measured were not molecule or formulation specific (Table 1). Differences in stability on the plates compared to the vials were assessed using rCE-SDS and SEC for all samples, and for a select subset of samples, MAM for PQAs and new peak detection and FlowCam for subvisible particle analysis.

**Figure 7:**
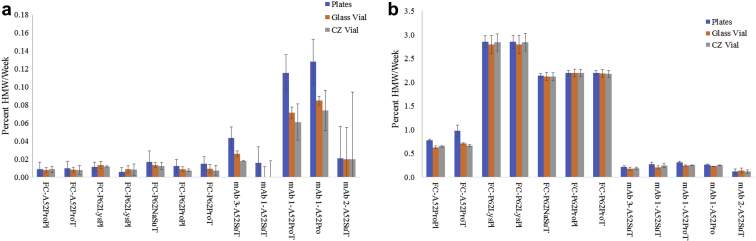
Rate of HMW formation in CZ plates, glass vials, and CZ vials over 12 weeks at (a) 4°C and (b) 40°C; *n* = 5 for the plate, and error bars are the standard error in HMW rate.

When the overall rate of HMW formation is compared across the 3 different container types, the overall trends are comparable. Figure 7a shows the rates at 4°C with all rates being comparable across the containers except for mAb3 in A52SuT and mAb1 in A52Pro and A52ProT. In these specific cases, the plates are slightly higher than the 2 comparable vial rates at 4°C. However, this is a very small difference (0.12 %HMW/wk compared to 0.07 %HMW/wk for the plate versus glass vial, respectively, for mAb1 in A52ProT). Figure 7b shows the rate of HMW formation at 40°C, a temperature that causes a more measurable change in the HMW. Again, the overall trends are comparable across the 3 different container types and across both low and high producing HMW formulations. For the Fc-fusion protein in A52ProPl and A52ProT, the plates have a slightly higher rate of HMW formation, with the biggest difference being a rate of 0.97 %HMW/wk for the plate compared to 0.71 % HMW/wk for the glass vial, comparable to the CZ vial. A noticeable difference was measured in these 2 formulations at the 12-week time point, with the plate having more HMW than the vials (Supplementary Fig. 1). The greater HMW in the plate is consistent with the higher measured evaporative loss at this time point which can cause more aggregation. If only the 12-week time point was being evaluated, the evaporative loss and variation in the plate versus the vials could provide misleading data. However, by calculating the rate of change over all 4 time points (T0-12 weeks), the effect of error in the final time point is reduced.

**Figure 8:**
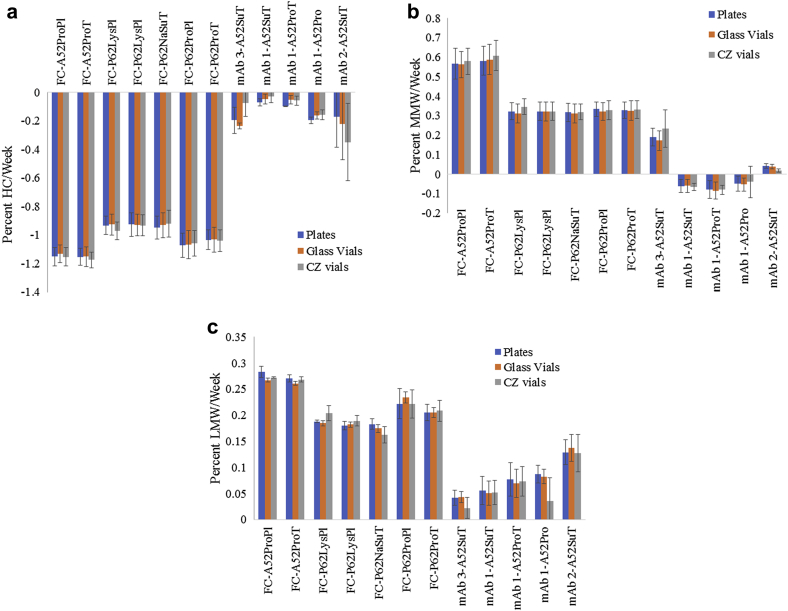
Rate of (a) HC or main, (b) MMW, and (c) LMW formation over 12 weeks at 40°C for CZ plates, glass vials, and CZ vials; *n* = 5 for the plate, and error bars are the standard error in rate.

Based on the calculated rate and standard error in rate, there was no statistical difference for the rate of change as measured by rCE-SDS for percent HC for the mAbs or percent main for the Fc-fusion protein, MMW, and LMW at 4°C between the CZ plate, CZ vial, and glass vial (Supplementary Fig. 2). In most formulations, the calculated standard error in rate was greater than the average rate itself. At 40°C, a more appreciable rate is measured and is shown in Figure 8. The overall trends are comparable across the 3 configurations for all formulations and molecules studied. In most cases, there is no statistically significant difference (based on the rate and standard error in rate) among the 3 configurations for each formulation.

As an additional test, MAM analysis was conducted at the 8- and 12-week time point at both temperatures for select formulations (Table 1) to evaluate any differences in common PQAs based on container type. Common modifications including glycosylation, glycation, isomerization, deamidation, and oxidation were evaluated for each sample at each time point. At any evaluated temperature and time point, no significant differences were observed for any modifications between the CZ plate, the CZ vial, and the glass vials. The greatest variation was measured for oxidation at Unk:192 in P62LysPl after 12 weeks at 40°C (Supplementary Table 5). The average across the containers at this condition was 11.72% ± 1.1%. As reported by Rogers et al.,^23^ this variability is within the precision-repeatability for oxidations, less than 11% of the measured value. As expected, increases in the relative amount for glycation, isomerization, deamidation, and oxidation were observed when the samples were compared at 4°C versus 40°C and across time points. A sample of the collected data can be found in the Supplementary Tables 1-5. An internal numbering scheme based on Honegger’s numbering scheme^32^ was used to name the structural location of the modifications. New peak detection was used to ensure that all potential changes in product quality were detected. No new peaks were detected in any formulation compared across the 3 containers at the same temperature and time point.

**Figure 9:**
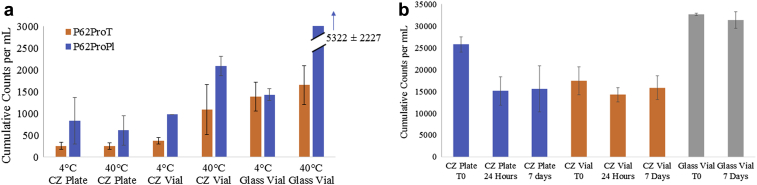
Cumulative counts per mL greater than or equal to 2 μm particle diameter in (a) the CZ plate (*n* = 2), CZ vial (*n* = 3), and glass vial (*n* = 3) after 12 weeks on stability for the Fc-fusion protein formulated in P62ProT and P62ProPl and (b) the CZ plate, CZ vial, and glass vial for mAb 3 over 7 days at room temperature (*n* = 3).

Subvisible particle analysis was conducted on the Fc-fusion protein in P62ProT and P62ProPl at the 12-week time point. As shown in Figure 9a, there was not a measurable difference for the CZ plates between the 4°C and 40°C temperatures for both formulations. However, a detectable increase in particle levels for the 2 vial types was measured at 40°C from 4°C. Notably, the CZ plate at 40°C had lower particle counts than the 2 vial types. The CZ vials were also trending lower than the glass vials at the elevated temperature. To explore whether particles were sticking to the vials or plates over time, a particulated mAb3 solution was aliquoted into a CZ plate, a CZ vial, and a glass vial and measured over the period of 1 week. As shown in Figure 9b, there is a decrease in the particle counts greater than or equal to 2 μm in diameter for the CZ plate from 25,779 ± 1774 particles/mL at time zero to 15,122 ± 3273 particles/mL at 24 h. This particle level was then maintained out to the 7-day time point. There is no difference in the particle counts across time for the CZ or glass vials.

Multiple scenarios may be responsible for the observation of reduced particles in the plate and are described here. First, based on the 12-week study (Fig. 9a) and the 1-week study (Fig. 9b), it is possible that particles could be adhering to the CZ plates more than to the glass and CZ vials. For the 12-week study, the surface area-to-volume ratio of the vials as compared to the plates is approximately 60%. For the 1-week study, the difference between the plate and vials is even greater with the surface area-to-volume ratio of the vials being 25% of the plate ratio. This greater surface area-to-volume ratio in the plate could possibly explain why more particles are adhering in the plate versus the vials. Alternatively, nucleation events leading to particle formation may be more likely to occur at glass rather than a CZ surface, leading to higher rates of subvisible particle formation. As shown in Figure 9a, the CZ vials trended lower than the glass vials on stability. A search of the literature did not report any studies in which subvisible particle counts decrease over time in CZ vials. However, there is a study that shows that CZ vials had less turbidity than glass vials when the same mAbs were exposed to mechanical agitation.^33^ Future studies are planned to study this trend in more detail, comparing the 3 configurations to determine whether more particles are adhering to the CZ plate compared to the vials and whether it is formulation or molecule dependent. The difference measured between the particle counts at time zero across the configurations in Figure 9b is thought to be due to mixing. A large volume of source material was used, and a constant, homogenous particle distribution was difficult to maintain. It is believed that the glass vial is higher in particle counts due to particles settling toward the bottom when it was aliquoted for this study. In a repeat experiment of time zero, the glass vial had lower particle counts than the CZ vial at time zero (data not shown).

## Conclusions

The CZ plates were evaluated for high-throughput formulation stability screening by studying evaporative loss effects and edge effects and comparing stability results from traditional glass and CZ vials to the CZ plates. It was found that minimal evaporation from the plates could be achieved at elevated temperatures with the use of a mylar bag and heat seal, and minimal evaporation occurred over time at and below 4°C. When the plates were examined for edge effects as measured by rCE-SDS and SEC, no noticeable effects were measured at the 4- and 8-week time point at 40°C but were measurable after 12 weeks of storage at 40°C. These effects were potentially due to evaporative loss and poor protein mixing in the wells. As such, precautions such as using multiple sample replicates or limiting samples to the inner 32 wells of a CZ plate should be used for studies going out to or past the 12-week time point at 40°C. Most importantly, when the stability for multiple molecules and formulations in CZ plates was compared to stability in CZ and glass vials, the relative order of stability remained the same. While there were some changes in the absolute measurement based on container, the overall ranking was comparable. When the plates were further interrogated by MAM, no difference among the 3 configurations was measured. A potential difference was measured between the 3 configurations for the subvisible particle counts. On stability, the CZ plates had less particles than the 2 vial configurations. The CZ plates also had a reduction in the particle count over time when stored at room temperature, potentially due to particles adhering to the CZ material in the plates. This was not demonstrated in the CZ or glass vials, but further studies are necessary to fully understand whether, how, and to what extent the vials or plate is impacting the subvisible particle count.

As a whole, these results indicate that the CZ plates could be used for high-throughput formulation stability screening if the proper controls are in place. At temperatures 4°C and potentially below 4°C, all wells of the CZ plate could be used with limited replicates to control for variation/error. However, at elevated temperatures, more replicates should be used, and the end time point should be carefully selected so that evaporative losses do not influence the results. These recommendations are made in the context of using the CZ plates as a screening assay and then verifying the final configuration of glass or CZ vials for the lead formulation.

The ability to use these microtiter plates for formulation stability studies enables lower material demands due to the smaller-volume wells and more formulation conditions to be tested. The physical footprint of a 96-well plate is also much smaller than a 96-vial storage rack, enabling a greater sample storage density. Furthermore, the plate format allows for easier automation integration. Automation can easily be used with the plates to aliquot out samples for SEC analysis. Alternatively, the CZ plate could be placed directly in an HPLC unit for measurement. In this study, automated sample preparation was used for diluting the samples for rCE-SDS analysis. It could also be further used for the rest of the preparation including addition of β-mercaptoethanol, incubating, and diluting with water. Sample preparation for other common formulation assays such as nonreduced-sodium dodecyl sulfate capillary electrophoresis, cation-exchange chromatography, and MAM could also be automated using the CZ plate as a “source” plate. Subvisible particle analysis was problematic and requires additional studies to understand the differences between the plate and vials. Additional characterization such as visible particle formation could be implemented by using specialized instrumentation, such as plate-based imaging.^34^ Other opportunities are enabled by the use of plates such as automated buffer exchange for studying multiple formulations and automated preparation of the samples in a sterile hood (both sterile filtration and aliquoting). Combining all of these approaches could shorten the amount of hands-on involvement that is required for each stage along with a shortening of timelines for the preparation, enabling a faster, high-throughput formulation screen.

## Supplementary Data

Supplementary Figure 1 Averaged HMW for CZ Plates (*n* = 5), glass vials (*n* = 1), and CZ vials (*n* = 1) at (a) 4 weeks at 4°C, (b) 4 weeks at 40°C, (c) 8 weeks and 4°C, (d) 8 weeks at 40°C, (e) 12 weeks at 4°C, and (f) 12 weeks at 40°C

Supplementary Figure 2 Rate of (a) HC or main, (b) MMW, and (c) LMW formation over 12 weeks at 4°C for CZ plates, glass vials, and CZ vials; *n* = 5 for the plate, and error bars are the standard error in rate
