# Supplementary figures and images for: Evaluation of Crystal Zenith Microtiter Plates for High-Throughput Formulation Screening
Source: J Pharm Sci. 2020 Jan;109(1):532–42. doi: 10.1016/j.xphs.2019.10.027 (PMC6941220; doi:10.1016/j.xphs.2019.10.027)

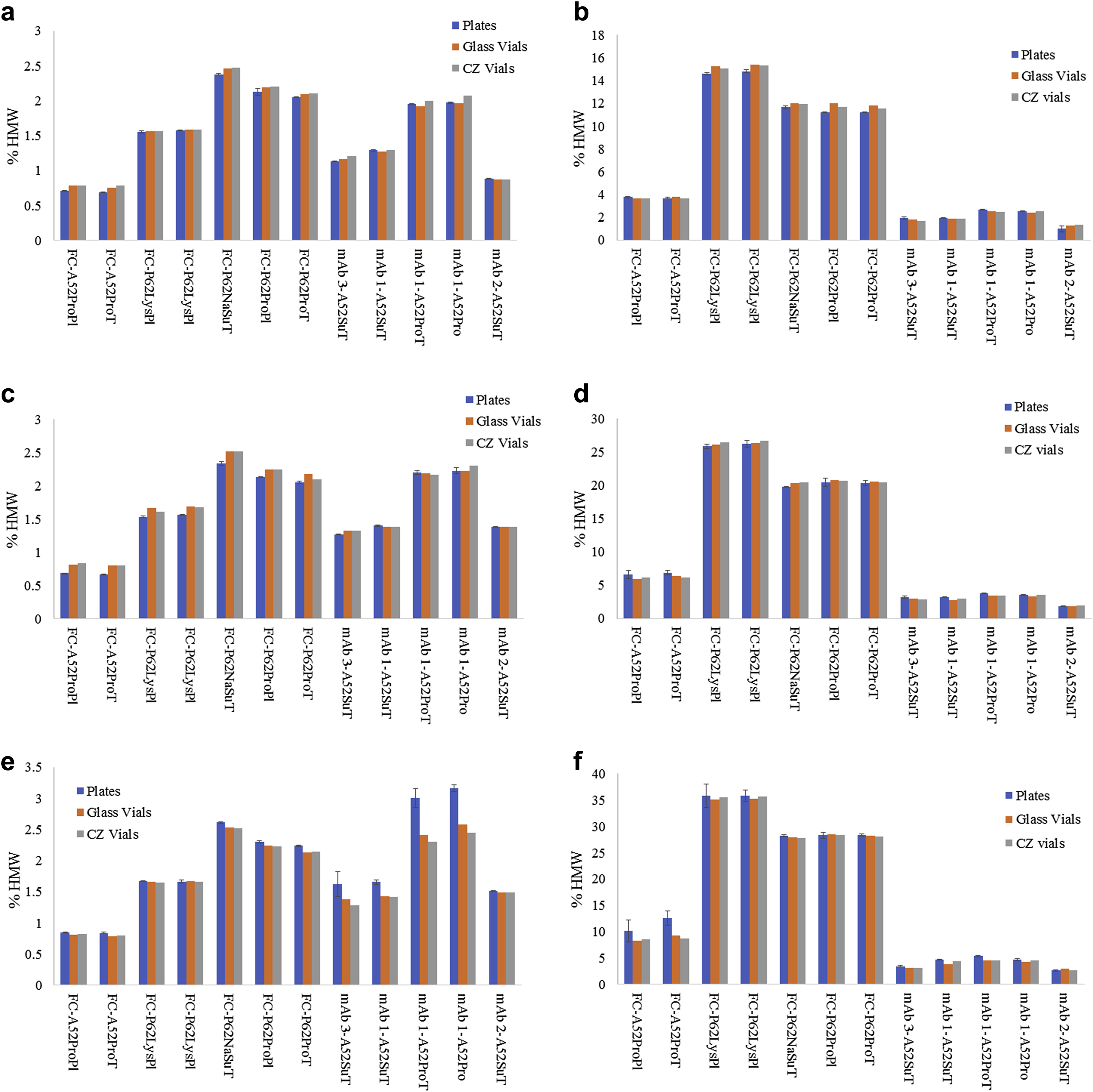

Supplement: Supplementary Figure 1 — Averaged HMW for CZ Plates (n = 5), glass vials (n = 1), and CZ vials (n = 1) at (a) 4 weeks at 4°C, (b) 4 weeks at 40°C, (c) 8 weeks and 4°C, (d) 8 weeks at 40°C, (e) 12 weeks at 4°C, and (f) 12 weeks at 40°C [file figs1.jpg]

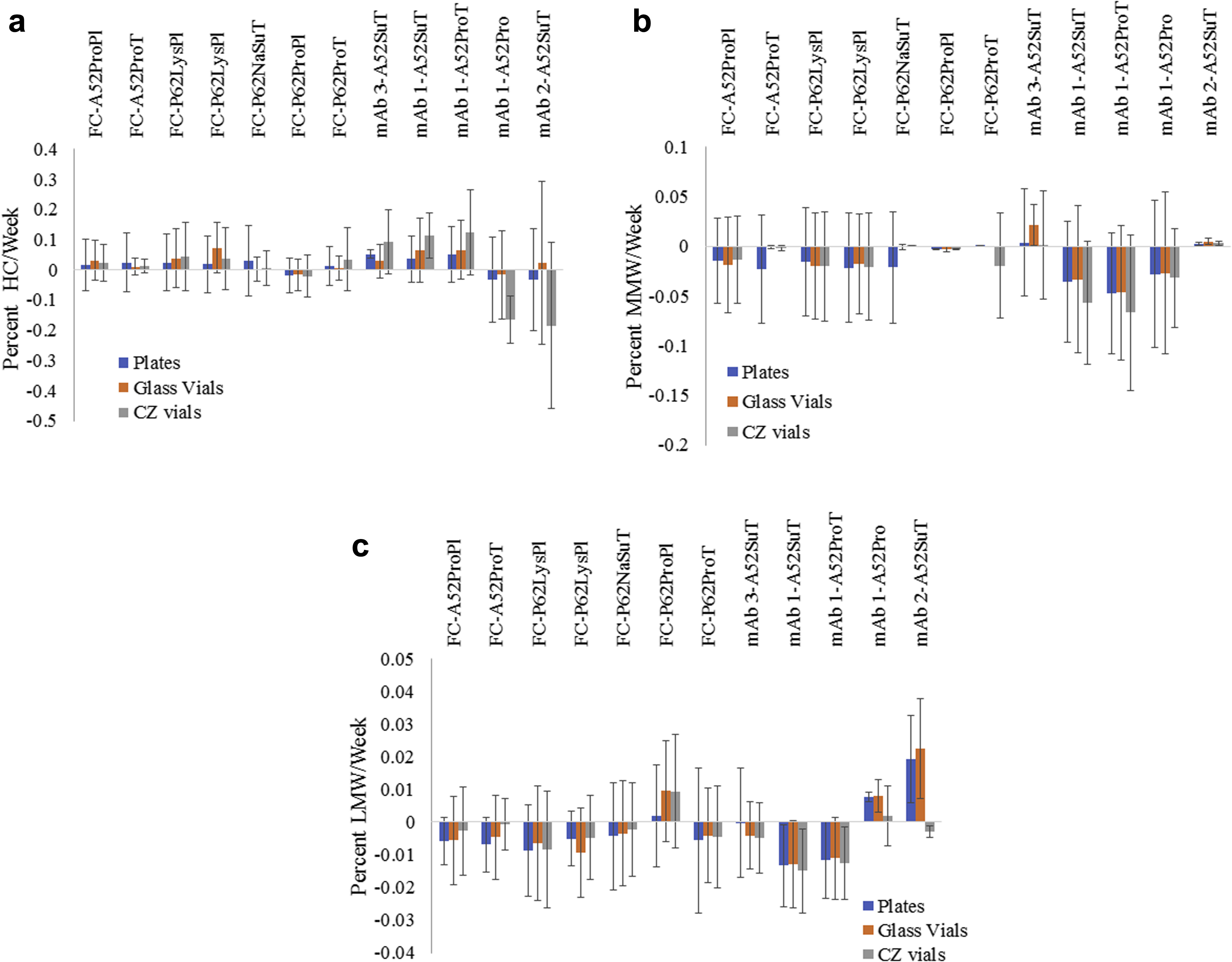

Supplement: Supplementary Figure 2 — Rate of (a) HC or main, (b) MMW, and (c) LMW formation over 12 weeks at 4°C for CZ plates, glass vials, and CZ vials; n = 5 for the plate, and error bars are the standard error in rate [file figs2.jpg]
