# Supplementary material for: Evaluation of Crystal Zenith Microtiter Plates for High-Throughput Formulation Screening
Source: J Pharm Sci. 2020 Jan;109(1):532–42. doi: 10.1016/j.xphs.2019.10.027 (PMC6941220; doi:10.1016/j.xphs.2019.10.027)
Supplement: Supplementary Table 1-5 [file mmc1.doc]

**Supplementary Table 1:** Selection of representative data collected by MAM for mAb1. Modifications are from both the Variable and Constant regions and include oxidation at methionine and tryptophan residues, isomerization (Iso.), and deamidation.

| Protein Name | Time Point | Condition | HV:83 Iso. | Fc-N:35 Iso. | KV:31 Deamidation | KV:4 Oxidation | W HV:82.4 Oxidation |
| --- | --- | --- | --- | --- | --- | --- | --- |
| mAb1, A52ProT | 8 Week | Time Zero Control | 0.01 | 0.00 | 0.45 | 0.46 | 0.48 |
|  |  | 4°C, CZ vial | 0.01 | 0.00 | 0.48 | 0.55 | 0.47 |
|  |  | 4°C, Glass vial | 0.01 | 0.00 | 0.44 | 0.51 | 0.43 |
|  |  | 4°C, CZ plate | 0.01 | 0.00 | 0.46 | 0.50 | 0.42 |
|  |  | 40°C, CZ vial | 0.62 | 0.65 | 1.38 | 1.14 | 0.68 |
|  |  | 40°C, Glass vial | 0.59 | 0.58 | 1.31 | 1.19 | 0.67 |
|  |  | 40°C, CZ plate | 0.55 | 0.54 | 1.35 | 1.38 | 0.70 |
|  | 12 Week | Time Zero Control | 0.01 | 0.00 | 0.44 | 0.60 | 0.48 |
|  |  | 4°C, CZ vial | 0.01 | 0.00 | 0.46 | 0.62 | 0.47 |
|  |  | 4°C, Glass vial | 0.01 | 0.00 | 0.46 | 0.61 | 0.50 |
|  |  | 4°C, CZ plate | 0.01 | 0.00 | 0.44 | 0.63 | 0.48 |
|  |  | 40°C, CZ vial | 1.02 | 1.29 | 1.68 | 1.34 | 0.85 |
|  |  | 40°C, Glass vial | 0.95 | 1.48 | 1.84 | 1.44 | 0.83 |
|  |  | 40°C, CZ plate | 0.95 | 1.22 | 1.81 | 1.63 | 1.02 |

**Supplementary Table 2:** Selection of representative data collected by MAM for mAb2. Modifications include oxidation, isomerization, glycation, and deamidation.

| Protein Name | Time Point | Condition | Fc-N:53 Isomerization | Fc-C:51 Deamidation | Fc-C:104 Oxidation | Fc-N:106 Glycation |
| --- | --- | --- | --- | --- | --- | --- |
| mAb2, A52SuT | 8 Week | Time Zero Control | 0.12 | 2.38 | 1.03 | 0.46 |
|  |  | 4°C, CZ vial | 0.12 | 2.62 | 1.05 | 0.48 |
|  |  | 4°C, Glass vial | 0.12 | 2.37 | 0.97 | 0.47 |
|  |  | 4°C, CZ plate | 0.11 | 2.59 | 1.05 | 0.49 |
|  |  | 40°C, CZ vial | 1.79 | 4.52 | 1.95 | 0.79 |
|  |  | 40°C, Glass vial | 1.82 | 4.58 | 2.80 | 0.75 |
|  |  | 40°C, CZ plate | 1.80 | 4.56 | 2.11 | 0.77 |
|  | 12 Week | Time Zero Control | 0.11 | 3.22 | 1.07 | 0.52 |
|  |  | 4°C, CZ vial | 0.11 | 3.30 | 1.28 | 0.51 |
|  |  | 4°C, Glass vial | 0.11 | 3.19 | 1.03 | 0.53 |
|  |  | 4°C, CZ plate | 0.11 | 3.26 | 1.21 | 0.53 |
|  |  | 40°C, CZ vial | 2.65 | 6.18 | 2.64 | 1.29 |
|  |  | 40°C, Glass vial | 2.61 | 6.27 | 3.54 | 1.23 |
|  |  | 40°C, CZ plate | 2.66 | 6.03 | 2.74 | 1.30 |

**Supplementary Table 3:** Selection of representative data collected by MAM for mAb3. Modifications are from both the Variable and Constant regions and include oxidation, isomerization (Iso.), glycation, and deamidation.

| Protein Name | Time Point | Condition | Fc-N:51 Iso. | HV:53 Iso. | Fc-C:51 Deamidation | HV:103 Oxidation | KV:144 Glycation |
| --- | --- | --- | --- | --- | --- | --- | --- |
| mAb3, A52SuT | 8 Week | Time Zero Control | 0.12 | 2.84 | 0.89 | 0.62 | 0.18 |
|  |  | 4°C, CZ vial | 0.13 | 3.43 | 0.90 | 0.65 | 0.12 |
|  |  | 4°C, Glass vial | 0.12 | 3.14 | 0.92 | 0.66 | 0.14 |
|  |  | 4°C, CZ plate | 0.13 | 3.09 | 0.91 | 0.56 | 0.15 |
|  |  | 40°C, CZ vial | 2.21 | 29.19 | 1.40 | 1.10 | 0.67 |
|  |  | 40°C, Glass vial | 2.17 | 29.05 | 1.39 | 1.09 | 0.70 |
|  |  | 40°C, CZ plate | 2.11 | 28.71 | 1.39 | 1.10 | 0.65 |
|  | 12 Week | Time Zero Control | 0.13 | 3.00 | 0.93 | 0.74 | 0.10 |
|  |  | 4°C, CZ vial | 0.13 | 3.13 | 0.87 | 0.75 | 0.10 |
|  |  | 4°C, Glass vial | 0.11 | 3.25 | 0.93 | 0.77 | 0.13 |
|  |  | 4°C, CZ plate | 0.13 | 3.05 | 0.87 | 0.78 | 0.14 |
|  |  | 40°C, CZ vial | 3.24 | 38.04 | 1.56 | 1.74 | 0.94 |
|  |  | 40°C, Glass vial | 3.08 | 38.15 | 1.55 | 1.40 | 0.86 |
|  |  | 40°C, CZ plate | 3.23 | 37.59 | 1.59 | 1.53 | 0.93 |

**Supplementary Table 4:** Selection of representative data collected by MAM for the Fc-fusion protein in P62NaSuT. Modifications include oxidation, isomerization (Iso.), and deamidation. The term Unk is used as the domain is not defined as an antibody region using the internal numbering scheme.

| Protein Name | Time Point | Condition | Unk:2 Iso. | Unk:84 Deamidation | Fc-N:59 Deamidation | Unk:192 Oxidation | Unk:140 Oxidation |
| --- | --- | --- | --- | --- | --- | --- | --- |
| Fc-fusion, P62NaSuT | 8 Week | Time Zero Control | 0.07 | 1.07 | 0.09 | 7.33 | 0.17 |
|  |  | 4°C, CZ vial | 0.07 | 1.19 | 0.12 | 7.21 | 0.16 |
|  |  | 4°C, Glass vial | 0.07 | 1.21 | 0.11 | 6.67 | 0.18 |
|  |  | 4°C, CZ plate | 0.07 | 1.16 | 0.12 | 6.84 | 0.19 |
|  |  | 40°C, CZ vial | 0.93 | 5.54 | 0.37 | 7.74 | 0.25 |
|  |  | 40°C, Glass vial | 0.89 | 5.66 | 0.36 | 6.57 | 0.22 |
|  |  | 40°C, CZ plate | 0.86 | 5.46 | 0.36 | 7.94 | 0.22 |
|  | 12 Week | Time Zero Control | 0.11 | 1.32 | 0.15 | 7.89 | 0.20 |
|  |  | 4°C, CZ vial | 0.10 | 1.41 | 0.18 | 8.92 | 0.21 |
|  |  | 4°C, Glass vial | 0.11 | 1.41 | 0.18 | 8.12 | 0.21 |
|  |  | 4°C, CZ plate | 0.10 | 1.37 | 0.17 | 8.13 | 0.22 |
|  |  | 40°C, CZ vial | 1.40 | 8.31 | 0.66 | 11.82 | 0.32 |
|  |  | 40°C, Glass vial | 1.36 | 8.50 | 0.62 | 10.35 | 0.32 |
|  |  | 40°C, CZ plate | 1.35 | 8.84 | 0.60 | 10.48 | 0.32 |

**Supplementary Table 5:** Selection of representative data collected by MAM for the Fc-fusion protein in P62LysPl. Modifications include oxidation, isomerization (Iso.), and deamidation. The term Unk is used as the domain is not defined as an antibody region using the internal numbering scheme.

| Protein Name | Time Point | Condition | Unk:2 Iso. | Unk:84 Deamidation | Fc-N:59 Deamidation | Unk:192 Oxidation | Unk:140 Oxidation |
| --- | --- | --- | --- | --- | --- | --- | --- |
| Fc-fusion, P62LysPl | 8 Week | Time Zero Control | 0.09 | 1.26 | 0.08 | 7.23 | 0.20 |
|  |  | 4°C, CZ vial | 0.08 | 1.33 | 0.09 | 7.48 | 0.19 |
|  |  | 4°C, Glass vial | 0.09 | 1.24 | 0.08 | 8.13 | 0.19 |
|  |  | 4°C, CZ plate | 0.08 | 1.27 | 0.09 | 7.55 | 0.20 |
|  |  | 40°C, CZ vial | 0.96 | 7.31 | 0.40 | 10.14 | 0.28 |
|  |  | 40°C, Glass vial | 0.96 | 7.34 | 0.40 | 9.94 | 0.30 |
|  |  | 40°C, CZ plate | 0.94 | 6.81 | 0.36 | 9.35 | 0.28 |
|  | 12 Week | Time Zero Control | 0.13 | 1.40 | 0.11 | 8.41 | 0.21 |
|  |  | 4°C, CZ vial | 0.13 | 1.69 | 0.14 | 8.81 | 0.21 |
|  |  | 4°C, Glass vial | 0.13 | 1.45 | 0.12 | 8.48 | 0.22 |
|  |  | 4°C, CZ plate | 0.12 | 1.62 | 0.12 | 8.33 | 0.21 |
|  |  | 40°C, CZ vial | 1.49 | 10.41 | 0.64 | 12.96 | 0.35 |
|  |  | 40°C, Glass vial | 1.53 | 10.45 | 0.61 | 11.23 | 0.40 |
|  |  | 40°C, CZ plate | 1.50 | 10.86 | 0.63 | 10.96 | 0.32 |
